# Incidence of subsequent malignancies after total body irradiation-based allogeneic HSCT in children with ALL – long-term follow-up from the prospective ALL-SCT 2003 trial

**DOI:** 10.1038/s41375-022-01693-z

**Published:** 2022-09-12

**Authors:** Anna Eichinger, Ulrike Poetschger, Evgenia Glogova, Peter Bader, Oliver Basu, Rita Beier, Birgit Burkhardt, Carl-Friedrich Classen, Alexander Claviez, Selim Corbacioglu, Hedwig E. Deubzer, Johann Greil, Bernd Gruhn, Tayfun Güngör, Kinan Kafa, Jörn-Sven Kühl, Peter Lang, Bjoern Soenke Lange, Roland Meisel, Ingo Müller, Martin G. Sauer, Paul-Gerhardt Schlegel, Ansgar Schulz, Daniel Stachel, Brigitte Strahm, Angela Wawer, Christina Peters, Michael H. Albert

**Affiliations:** 1 Department of Pediatrics, Dr. von Hauner Children’s Hospital, University Hospital, LMU, Munich, Germany; 2 Children’s Cancer Research Institute, Vienna, Austria; 3 Division for Stem Cell Transplantation and Immunology, Department for Children and Adolescents, University Hospital, Goethe University, Frankfurt/Main, Germany; 4 Department of Pediatrics III, University Hospital Essen, University Medicine Essen, Essen, Germany; 5 Department of Pediatric Oncology and Hematology, Medizinische Hochschule Hannover, Hannover, Germany; 6 Pediatric Hematology and Oncology, University Hospital Muenster, Muenster, Germany; 7 Oncology and Hematology Unit, Children’s Hospital, University Medicine Rostock, Rostock, Germany; 8 Department of Pediatrics and Bone Marrow Transplant Unit, Medical University of Schleswig-Holstein, Campus Kiel, Kiel, Germany; 9 Department of Pediatric Hematology, Oncology and Stem Cell Transplantation, University of Regensburg, Regensburg, Germany; 10 Department of Pediatric Hematology and Oncology, Charité – Universitätsmedizin Berlin, Corporate Member of Freie Universität Berlin and Humboldt-Universität Berlin, Berlin, Germany; 11 Experimental and Clinical Research Center (ECRC) of the Charité and the Max-Delbrück-Center for Molecular Medicine (MDC) in the Helmholtz Association, Berlin, Germany; 12 Department of Pediatric Oncology, Hematology and Immunology, University of Heidelberg, Heidelberg, Germany; 13 Department of Pediatrics, Jena University Hospital, Jena, Germany; 14 Department of Hematology/Oncology/Immunology, Gene-therapy, and Stem Cell Transplantation, University Children’s Hospital Zurich – Eleonore Foundation & Children’s Research Center (CRC), Zurich, Switzerland; 15 Department of Pediatrics, University of Halle, Halle, Germany; 16 Department of Pediatric Oncology, Hematology and Hemostaseology, University of Leipzig, Leipzig, Germany; 17 University Children’s Hospital, Tübingen, Germany; 18 Department of Pediatrics, University Hospital Carl Gustav Carus, Technical University of Dresden, Dresden, Germany; 19 Division of Pediatric Stem Cell Therapy, Department of Pediatric Oncology, Hematology and Clinical Immunology, Medical Faculty, Heinrich-Heine-University, Düsseldorf, Germany; 20 Divison of Pediatric Stem Cell Transplantation and Immunology, Clinic for Pediatric Hematology and Oncology, University Medical Center Hamburg-Eppendorf, Hamburg, Germany; 21 Department of Pediatric Hematology and Oncology, University Children’s Hospital Würzburg, Würzburg, Germany; 22 Department of Pediatrics, University Medical Center Ulm, Ulm, Germany; 23 Children’s Hospital, Universitätsklinikum Erlangen, Erlangen, Germany; 24 Division of Pediatric Hematology and Oncology, Department of Pediatrics and Adolescent Medicine, Medical Center, Faculty of Medicine, University of Freiburg, Freiburg, Germany; 25 Department of Pediatrics and Children’s Cancer Research Center, Kinderklinik München Schwabing, Technical University of Munich School for Medicine, Munich, Germany; 26 St. Anna Children’s Hospital, University Vienna, St. Anna Children’s Cancer Research Institute, Vienna, Austria

**Keywords:** Acute lymphocytic leukaemia, Medical research

## Abstract

Total body irradiation (TBI)-based conditioning is associated with superior leukemia-free survival in children with ALL undergoing HSCT. However, the risk for subsequent malignant neoplasms (SMN) remains a significant concern. We analyzed 705 pediatric patients enrolled in the prospective ALL-SCT-BFM-2003 trial and its subsequent registry. Patients >2 years received conditioning with TBI 12 Gy/etoposide (*n* = 558) and children ≤2 years of age or with contraindications for TBI received busulfan/cyclophosphamide/etoposide (*n* = 110). The 5- and 10-year cumulative incidence of SMN was 0.02 ± 0.01 and 0.13 ± 0.03, respectively. In total, 39 SMN (34 solid tumors, 5 MDS/AML) were diagnosed in 33 patients at a median of 5.8 years (1.7–13.4), exclusively in the TBI group. Of 33 affected patients, 21 (64%) are alive at a median follow-up of 5.1 years (0–9.9) after diagnosis of their first SMN. In univariate analysis, neither age at HSCT, donor type, acute GVHD, chronic GVHD, nor CMV constituted a significant risk factor for SMN. The only significant risk factor was TBI versus non-TBI based conditioning. This analysis confirms and quantifies the increased risk of SMN in children with ALL after conditioning with TBI. Future strategies to avoid TBI will need careful tailoring within prospective, controlled studies to prevent unfavorable outcomes.

## Introduction

The long-term overall survival (OS) rate of children with acute lymphoblastic leukemia (ALL) has improved to more than 80% over the past decades [1–4]. High-risk cases with an indication for allogeneic hematopoietic stem cell transplantation (HSCT) can expect OS rates of 50–90% [5–7]. Thus, negative long-term effects of HSCT and their management are increasingly the focus of attention when evaluating treatment choices for children with ALL.

With current therapies, a large proportion of pediatric cancer survivors have one or more long-term adverse effects after HSCT [8, 9]. While the risk of relapse-related death after HSCT plateaus, non-relapse related causes of death—including subsequent malignant neoplasms (SMN)—continue to accumulate over time [10, 11]. The emergence of SMN is a particular gruesome side effect of HSCT with significant mortality. Due to its unpredictability, the fear of SMN is adding to the state of uncertainty of leukemia survivors which can lead to psychosocial problems [12, 13].

Total body irradiation (TBI) in various doses and fractioning schemes has been used in the past decades in HSCT conditioning protocols. Major advantages of TBI are its potent anti-leukemic activity even in organs not easily reached by systemic chemotherapy (e.g. testes, brain) and its strong immunosuppressive effect [14, 15]. TBI has repeatedly been reported as a risk factor for the development of SMN [16]. Therefore, TBI-based conditioning has been abandoned for nearly all pediatric HSCT indications except for ALL. The prospective ALL-SCT-BFM 2003 trial (Allogeneic Stem Cell Transplantation in Children and Adolescents with Acute Lymphoblastic Leukemia, NCT01423747) demonstrated excellent survival rates using a uniform TBI/etoposide (VP-16) conditioning in children >2 years and no differences in overall survival (OS), event-free survival (EFS), and cumulative incidence of relapse between those children who received a transplant from matched sibling donors (MSD) and matched unrelated donors (MUD) [6]. The age cut-off at 2 years was historically driven in Germany, Austria and Switzerland. In the subsequent multi-national, prospective, randomized FORUM trial the lower age limit was set at 4 years of age for reasons of conformity with standard practice in other regions. The FORUM trial aimed to demonstrate non-inferiority of a potentially less toxic chemotherapy-based HSCT conditioning compared to TBI/VP-16 in children with ALL. However, the randomization was prematurely abandoned because of a significantly higher 2-year-cumulative incidence of relapse in the chemotherapy arm [5].

TBI-based conditioning clearly results in superior leukemia free survival and significant lower treatment related mortality compared to chemo-conditioning in children with ALL, but prospective studies of its role in the development of SMN in this age group are lacking (Supplementary Table 1). Therefore, we analyzed the incidence, outcome, and risk factors for SMN in the prospective ALL-SCT-BFM 2003 trial and its subsequent extension registry.

## Methods

### Study protocol

This analysis is part of the prospective multicenter ALL-SCT-BFM-2003 trial (September 2003 to September 2011) and its subsequent extension registry (October 2011 to September 2013). The trial protocol is described in detail elsewhere [6]. The study protocol was approved by the local institutional review board at each participating site. Patients and/or their legal guardians provided written informed consent before enrolment. This study was performed in accordance with the Declaration of Helsinki for Good Clinical Practice and was registered at www.clinicaltrials.gov (NCT01423747). Data analysis was performed by EG and UP and all authors had access to primary clinical trial data.

Data from 705 patients were available for this analysis; 411 patients were transplanted as part of the trial and 294 additional patients in the extension registry. The indication for HSCT was determined by the stratification criteria of the frontline chemotherapy protocols. Briefly, these included patients in first complete remission (CR1) with induction failure, t(4;11), t(9;22), or very poor MRD response; patients in CR2 except those with late isolated extramedullary relapse; and patients with any CR > 2. Conditioning consisted of TBI (12 Gy in 6 fractions of 2 Gy, given as 2 fractions per day over 3 days) and VP-16 (60 mg/kg; upper total dose 3600 mg) in patients >2 years and without contraindications for TBI. Patients with contraindications for TBI (central nervous system (CNS) irradiation before HSCT, history of CNS toxicities, or signs of leukoencephalopathy) received chemotherapy conditioning according to protocol. Other reasons for not receiving TBI were logistic hurdles and/or patients/parents‘ refusal. For TBI, in vivo dosimetry accepting deviations of ±5% was recommended per protocol. Patients ≤2 years and children with contraindications for TBI were conditioned with busulfan (full myeloablative weight-based dose), cyclophosphamide (120 mg/kg total dose) and VP-16 (40 mg/kg total dose). Busulfan was administered according to weight-based dosing recommended by the manufacturer. Pharmacokinetic monitoring was not mandated by the study protocol. All histologically confirmed malignancies reported after HSCT were assessed. Post-transplant EBV-related lymphoproliferative disorders (PTLD) were not classified as SMN.

### Statistical analysis

Statistical analysis was performed by the ALL-SCT-BFM-2003 trial statistician (UP). OS and EFS probabilities were evaluated using the Kaplan-Meier method, taking the day of HSCT as the starting point for the calculation. For the estimation of EFS, the date of the first event (relapse, SMN or death of any cause) or the last examination date were taken as the end point of the time interval. The cumulative incidence of SMN was calculated by the method of Kalbfleisch and Prentice and compared using the Gray test [17, 18]. Death of any cause was defined as a competing event. For the purpose of the analysis of risk factors, the incidence of a SMN as a first event was calculated defining death of any cause and relapse as competing events.

The proportional subdistribution hazards model of Fine and Gray for censored data subject to competing risks was applied for the univariate analysis where appropriate. For the univariate analysis of the effect of TBI or age on the incidence of SMN, and the multivariate analysis of SMN incidence we applied the Cox proportion hazard model with Firth’s modification of the maximum likelihood estimation, because no SMN was observed in subsets (patients with no TBI and patients <2 years) [19, 20]. In the multivariate analysis, the effect of the following factors was studied for their potential association with the incidence of SMN: age, sex match, donor type, stem cell source, disease recurrence risk at time of HSCT, CMV constellation, TBI, and leukemia immunophenotype. The impact of chronic graft-versus-host-disease (GVHD) on the cumulative incidence of SMN was assessed separately by means of a Fine and Gray model after adjustment for the variables mentioned above, including chronic GVHD as a time dependent covariate.

For non-time-to-event variables the Chi Square test or, where appropriate, the Fisher exact test were used to compare groups for categorical variables, and the Wilcoxon rank-sum test was used for continuous variables. All *p*-values were two-sided, and those below 0.05 were considered significant. The statistical analyses were performed by means of the SAS version 9.4 (SAS Institute, Cary, NC).

### Data sharing statement

Deidentified, summarized original data are available upon written request to CP (christina.peters@stanna.at). Individual participant data will not be shared. The study protocol is included in the data supplement available with the online version of this article.

## Results

### Patients

A total of 705 patients were eligible for analysis (Table 1). The median follow-up after HSCT was 5.3 years (range: 0.01–16.4). The majority of children (*n* = 678; 96%) was older than two years of age at HSCT. Most patients were conditioned with TBI/VP-16 (*n* = 558; 79%), while *n* = 110 (16%) received chemo-conditioning (Bu/Cy/VP-16). Patient characteristics are depicted in Table 1. Information on conditioning regimen was not available for 37 patients (5%). The probability of OS was 0.70 ± 0.02 at 5 years and 0.64 ± 0.03 at 10 years. EFS was 0.64 ± 0.02 and 0.52 ± 0.04, respectively (Supplementary Fig. S1). Seventy patients (10%) died within the first 100 days after allogeneic HSCT, none of them was diagnosed with a SMN.

**Table 1:** Patient characteristics.

	*n* = 705^a^	TBI group (*n* = 558^a^)	Non-TBI group (*n* = 110^a^)	*p*-value^b^
Follow-Up				
Median Follow-up in years^c^		5.76	4.35	
Minimum in years		0.05	0.04	
Maximum in years		16.37	11.18	
Median Follow-Up (*patients alive*)		5.43	4.13	0.001
Age				
≤2 years	27 (4%)	0 (0%)	24 (22%)	<0.001
2–4 years	55 (8%)	39 (7%)	15 (14%)	
>4 years	623 (88%)	519 (93%)	71 (65%)	
Sex				
Male	433 (61%)	363 (65%)	59 (54%)	0.02
Female	249 (35%)	189 (34%)	51 (46%)	
Missing data	23 (3%)	6		
Donor type				
MSD	181 (26%)	150 (27%)	17 (15%)	<0.001
MD	438 (62%)	362 (65%)	59 (54%)	
MMD	86 (12%)	46 (8%)	34 (31%)	
Remission status				
CR1	333 (47%)	263 (47%)	58 (53%)	0.25
CR2	309 (44%)	249 (45%)	40 (36%)	
CR > 2	63 (9%)	46 (8%)	12 (11%)	
Stem cell source				
BM	456 (65%)	393 (71%)	52 (48%)	<0.001
PB	217 (31%)	157 (28%)	54 (50%)	
CB	12 (2%)	7 (1%)	3 (3%)	
Missing data	20 (3%)	1	1	
Acute GVHD				
0	172 (24%)	138 (25%)	29 (27%)	0.28
I	258 (37%)	221 (40%)	34 (32%)	
II	158 (22%)	132 (24%)	24 (22%)	
III	47 (7%)	34 (6%)	12 (11%)	
IV	19 (3%)	15 (3%)	4 (4%)	
Death without aGVHD	16 (2%)	12 (2%)	4 (4%)	
Missing data	35 (5%)	6	3	
Chronic GVHD				
None	406 (58%)	337 (68%)	57 (63%)	0.13
Limited	74 (10%)	62 (13%)	11 (12%)	
Extensive	56 (8%)	49 (10%)	6 (7%)	
Death prior d100	70 (10%)	48 (10%)	16 (18%)	
Missing data	99 (14%)	62	20	
CMV status (donor/recipient)				
−/−	283 (40%)	236 (45%)	44 (42%)	0.94
−/+	98 (14%)	79 (15%)	16 (15%)	
+/−	103 (15%)	85 (16%)	18 (17%)	
+/+	158 (22%)	128 (24%)	28 (26%)	
Missing data	63 (9%)	30	4	
Recipient CMV status				
Negative	397 (56%)	329 (62%)	64 (59%)	0.68
Positive	263 (37%)	212 (41%)	45 (41%)	
Missing data	45 (6%)	17	1	

*BM* bone marrow, *CB* cord blood, *CR* complete remission, *CMV* cytomegalovirus, *GVHD* graft-versus-host-disease, *MD* matched donor, *MMD* mismatched donor, *MSD* matched sibling donor, *n.a.* not available, *PB* peripheral blood.

^a^37 patients with missing information on TBI are not included in either column but are included in the general column.

^b^Chi Square test.

^c^Reverse Kaplan–Meier estimator.

### Subsequent malignancies

**Fig. 1:**
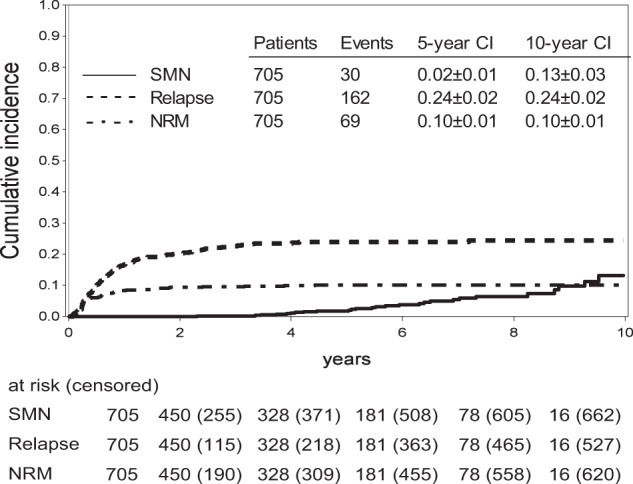
Cumulative incidences of severe events post HSCT: cumulative incidence of first subsequent malignancy neoplasm (SMN, solid line), non-relapse mortality (NRM, dashdotted line) and relapse (dashed line) after HSCT.

In total, 39 SMN were reported in 33 patients (5%, Fig. 1; Table 2). The 5-, 8- and 10-year cumulative incidences of SMN in this cohort (with relapse and non-relapse mortality as competing events) were 0.02 ± 0.01, 0.06 ± 0.01 and 0.13 ± 0.02, respectively (Fig. 1).

**Table 2:** Localization, Staging, Therapy and Follow-Up of Subsequent Malignancies (SMN).

Patient	Age at HSCT (in years)	Time to onset of first SMN after HSCT (in years)	First SMN		Initial therapy for SMN^b^	Time to onset of additional SMN after HSCT (in years)	Additional SMN		Initial therapy for additional SMN	Outcome		Follow-up (after first SMN in years)
			Type	Localization, Staging^a^			Type	Localization, Staging		Course of SMN	Patient alive at last follow-up (cause of death)	
1	5.5	8.3	Papillary thyroid carcinoma	pT3 N1a M0	Surgery, ablative iodine therapy	-	-	-	-	No relapse	Yes	5.7
2	4.0	3.7	Papillary thyroid carcinoma	n.a.	Surgery, ablative iodine therapy	-	-	-	-	No relapse	Yes	2.4
3	10.4	7.0	Papillary thyroid carcinoma	pT2 N1 M0	Surgery, ablative iodine therapy	-	-	-	-	No relapse	Yes	1.9
4	2.9	7.3	Papillary thyroid carcinoma	pT1b pN1a M0	Surgery, ablative iodine therapy	-	-	-	-	No relapse	Yes	6.0
5	3.8	6.3	Papillary thyroid carcinoma	pT1a pN1a Mx	Surgery (R1), ablative iodine therapy	-	-	-	-	No relapse	Yes	9.9
6	7.9	10.3	Papillary thyroid carcinoma	pT3a pN0	Surgery (left pRx/cR0, right R0), ablative iodine therapy	10.7	Squamous cell carcinoma	Left dorsal hand	Surgery	No relapse	Yes	5.9
7	7.5	4.2	Papillary thyroid carcinoma	pT2 pN1b	Surgery (R0), ablative iodine therapy	-	-	-	-	No relapse	Yes	6.4
8	15.6	3.4	Papillary thyroid carcinoma	pT1 pN1a M0	Surgery (R0), ablative iodine therapy	10.5	MDS RAEB-t/ AML (recipient type)	-	Allogeneic HSCT	No relapse of thyroid carcinoma	No (graft failure, adeno virus infection)	7.7
9	4.9	5.5	Papillary thyroid carcinoma	pT3 pN1 M0	Surgery (R0), ablative iodine therapy	-	-	-	-	No relapse	Yes	6.9
10	5.7	4.0	Papillary thyroid carcinoma	pT3 pN1a pMx	Surgery, ablative iodine therapy	-	-	-	-	No relapse	Yes	6.3
11	4.8	9.3	Papillary thyroid carcinoma	pT1b pN1b	Surgery (R0), ablative iodine therapy	-	-	-	-	No relapse	Yes	7.1
12	5.1	11.3	Papillary thyroid carcinoma	pT3 pN1a M0	Surgery (R0), ablative iodine therapy	-	-	-	-	No relapse	Yes	4.1
13	3.0	5.7	Papillary thyroid carcinoma	pT1a pN1a M0, R0	Surgery (R0)	-	-	-	-	No relapse	Yes	2.2
14	9.1	5.9	Papillary thyroid carcinoma	pT3 N1 M0	Surgery	-	-	-	-	No relapse	Yes	3.9
15	5.5	6.5	Glioblastoma	Glioblastoma multiforme, WHO °IV	Surgery, Radiotherapy, Chemotherapy	-	-	-	-	Tumor progression (glioblastoma)	No (SMN)	0.7
16	16.4	6.4	Glioblastoma	Glioblastoma multiforme, WHO °IV	Supportive palliative care	-	-	-	-	Tumor progression (glioblastoma)	No (SMN)	0.1
17	13.7	8.7	Glioblastoma	Glioblastoma multiforme, WHO °IV	Surgery, Radiotherapy, Chemotherapy	-	-	-	-	Tumor progression (glioblastoma)	No (SMN)	3.5
18	10.2	7.1	Osteosarcoma	Distal femur, M0	Surgery, chemotherapy	-	-	-	-	Osteosarcoma recurrence with lung metastases	No (SMN)	2.1
19	10.1	8.8	Osteosarcoma	Right distal radius, T1, N0, M0	Surgery, chemotherapy	-	-	-	-	No relapse	Yes	5.1
20	3.3	3.5	Osteosarcoma	thoracic vertebrae 5/6 and dorsolateral associated ribs; T3N0M0	Surgery, chemotherapy	-	-	-	-	No relapse	Yes	6.5
21	6.7	5.5	Basal cell carcinoma	Right cervical region (5 mm diameter)	Surgery (R0)	-	-	-	-	No relapse	Yes	9.6
22	10.9	9.5	Basal cell carcinoma	Back, left of the spinal cord	Surgery	12.0	Breast carcinoma	Left breast, NST, G2 pT1b(is) pN0 M0 L0 V	Mastectomy, anti-hormonal therapy	Recurrence breast carcinoma (treated with radiotherapy)	Yes	5.2
23	14.4	6.9	Basal cell carcinoma	Right shoulder (ventral) to region above right clavicle	Surgery	11.2	Basal cell carcinoma	Right thigh (dorsal)	Surgery	No relapse	Yes	6.8
24	5.1	3.4	Colon carcinoma	Low-grade intraepithelial neoplasia with transition to high grad intraepithelial neoplasia, N0 M0	Surgery	4.3	MDS	n.a. (donor/recipient origin unknown)	n.a.	MDS progression	No (bleeding)	2.2
25	9.3	3.9	Colon carcinoma	Adenocarcinoma (colon transversum and rectum), pT3 pN1 M0	Surgery (R0), Chemotherapy	10.7	Glioblastoma multiforme, WHO °IV	-	Surgery, radiotherapy, chemotherapy	Tumor progression (glioblastoma)	No (SMN)	7.6
26	13.5	1.8	Breast carcinoma	n.a.	n.a.	-	-	-	-	ALL relapse	No (ALL)	0.9
27	18.1	13.4	Squamous cell carcinoma	Right calf with bone involvement	Surgery	-	-	-	-	No relapse	Yes	0
28	10.3	2.3	Ewing sarcoma	Right lower jawbone	Surgery, chemotherapy	-	-	-	-	Ewing sarcoma recurrence with lung metastases	No (SMN)	3.3
29	8.4	5.2	Parotid carcinoma	Mucoepidermoid carcinoma of the right parotis, N0 M0	Surgery	-	-	-	-	No relapse	Yes	5.8
30	18.4	5.1	Rhabdomyosarcoma	Embryonal rhabdomyosarcoma, sole of the right foot, spinal metastasis	Supportive palliative care	-	-	-	-	Tumor progression (rhabdomyosarcoma)	No (SMN)	1.0
31	13.3	5.0	MDS/AML	donor/recipient origin unknown	Allogeneic HSCT	-	-	-	-	No relapse	Yes	5.5
32	4.4	4.1	MDS/AML	MDS RAEB-t/AML (donor type)	Allogeneic HSCT	-	-	-	-	Disease progression (MDS)	No (SMN)	4.0
33	17.2	4.5	MDS/AML	MDS (refractory cytopenia; donor/ recipient origin unknown))	Allogeneic HSCT	-	-	-	-	Multiorgan failure due to sepsis and chemotoxicity in preparation for third HSCT (for MDS)	No (therapy related)	2.6

*HSCT* hematopoietic stem cell transplantation, *n.a.* not available, *SMN* subsequent malignant neoplasm(s).

^a^TNM stadium.

^b^Resection status (R) after surgery, if available.

The centers reported five cases of myelodysplastic syndrome/AML (MDS/AML; 13%) and 34 cases of solid tumors (87%): thyroid cancer (*n* = 14; 36%), glioblastoma (*n* = 4; 10%), basal cell carcinoma (*n* = 4; 10%), osteosarcoma (*n* = 3; 8%), colon carcinoma (*n* = 2; 5%), breast cancer (*n* = 2; 5%), squamous cell carcinoma (*n* = 2; 5%), and Ewing sarcoma, parotid carcinoma and rhabdomyosarcoma (*n* = 1; 3% each). The MDS were of donor origin (*n* = 1), recipient origin (*n* = 1), or unknown origin (*n* = 3).

Six patients developed an additional SMN. These were MDS (*n* = 2), glioblastoma, breast cancer, basal cell and squamous cell carcinoma (one each). Three patients had experienced a prior ALL relapse at the time of diagnosis of SMN.

The first SMN occurred at a median of 5.7 years (1.7–13.4) post HSCT, and the second occurred at 10.7 years (4.3–12.1) after HSCT. MDS (*n* = 3 as first SMN) developed after 4.5 ± 0.5 years compared to solid tumors at 6.3 ± 2.7 years (*p* = 0.25).

The majority of patients (31 of 32 with information available) received specific anti-neoplastic treatment for their first SMN (data not available for one patient; 3%). SMN treatment included chemotherapy only (*n* = 1), surgery only (*n* = 8), surgery combined with chemotherapy (*n* = 5), allogeneic HSCT (*n* = 3), surgery combined with chemotherapy and radiation (*n* = 2), and surgery combined with ablative iodine therapy (*n* = 12). One patient with glioblastoma received primary supportive palliative care (Table 2).

**Fig. 2:**
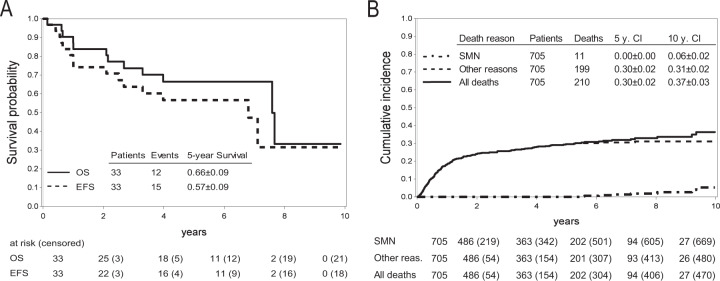
Survival after diagnosis of a subsequent malignancy (SMN), and cumulative incidence of death due to SMN. **A** Overall (OS, solid line) and event-free survival (EFS, dashed line; event = relapse of SMN or ALL, additional SMN, death) after diagnosis of first SMN. **B** Cumulative incidence of death: all deaths (solid line), death due to SMN (dashdotted line) and death due to another reason (dashed line).

Of the 33 affected patients, 21 (64%) are alive at a median follow-up of 5.8 years (0–9.9) after diagnosis of their first SMN (Fig. 2A). All patients diagnosed with glioblastoma died within ten months (1.7–9.6 months) of diagnosis. Almost all patients diagnosed with thyroid cancer (93%) were alive at last follow-up (0–9.9 years since diagnosis). One patient developed MDS as second SMN and died of graft failure and adenovirus infection with multiorgan failure after a second HSCT. The cumulative incidence of death due to a SMN was 0.00 ± 0.00 at 5 years and 0.06 ± 0.02 at 10 years for the entire cohort (Fig. 2B).

Systematic testing for cancer predisposition syndromes was not part of the study protocol. One patient who had developed two SMN (colon carcinoma and glioblastoma) and with suspicious family history was diagnosed with Lynch syndrome (*MSH6*).

### Risk factors

**Fig. 3:**
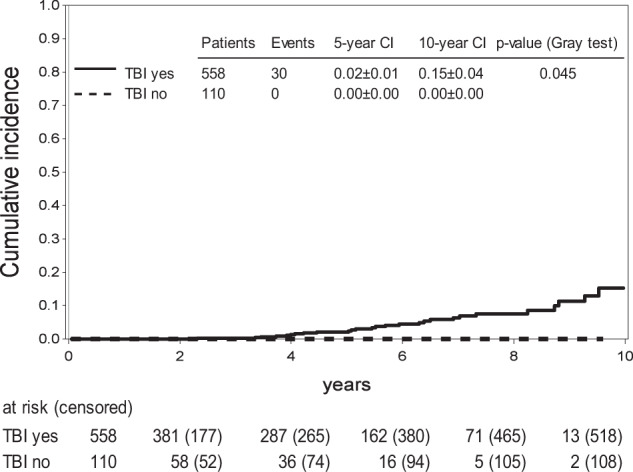
Cumulative incidence of subsequent malignancy (SMN) depending on conditioning regimen: Cumulative incidence of SMN in patients >2 years of age, comparing patients with TBI (total body irradiation) conditioning (solid line) with patients without TBI conditioning (dashed line).

In univariate analysis, neither age at HSCT, donor type, acute GVHD, chronic GVHD, stem cell source, donor type (MSD, MD, MMD), remission status at HSCT or CMV constellation constituted a significant risk factor for the development of a SMN (Table 3). SMN occurred exclusively in patients who received TBI/VP-16 conditioning. The 8-year cumulative incidence of SMN in this subset of patients was 0.07 ± 0.02 (Fig. 3). No child ≤2 years at HSCT (*n* = 27) and none of the other children (*n* = 83) who were conditioned with Bu/Cy/VP-16 or those with missing information about their conditioning regimen (*n* = 37) were affected by SMN. Thus, the cumulative incidence of a SMN was significantly increased in patients who had received TBI/VP-16 versus those without TBI (*p* = 0.045) (Fig. 3). Multivariate analysis was performed even though it is of restricted validity due to the low overall number of events. It did not identify any statistically significant risk factors (age, donor type, disease recurrence risk, stem cell source, CMV constellation, TBI versus no TBI, leukemia phenotype; Supplementary Table 2).

**Table 3:** Univariate analysis of risk factors for developing SMN.

risk factor	8 year cumulative incidence ± SD	hazard ratio [95% CI]	*p*-value^a^
Conditioning			
TBI	0.07 ± 0.02	6.31 [0.36, 109.54]	**0.045**
No TBI or missing	0.00 ± 0.00		
Age at HSCT			
≤2 yrs	0.00 ± 0.00	< 2 yrs vs. >10 yrs: 0.55 [0.03, 10.26]	0.12
2–10 yrs	0.08 ± 0.02	2 yrs–10 yrs vs. >10 yrs: 1.72 [0.80, 3.72]	
>10 yrs	0.05 ± 0.02		
Donor type			
MSD	0.05 ± 0.02	MSD vs. MMD: 3.83 [0.49, 30.13]	0.21
MD	0.09 ± 0.02	MD vs. MMD: 4.46 [0.59, 33.56]	
MMD	0.02 ± 0.02		
Acute GVHD			
0–II	0.07 ± 0.02	0.81 [0.27, 2.43]	0.67
III–IV	0.08 ± 0.06		
Chronic GVHD			
No	0.08 ± 0.02	1.256 [0.58, 2.70]	0.83
Yes	0.11 ± 0.04		
Remission status at HSCT			
CR1	0.08 ± 0.03	0.90 [0.44, 1.84]	0.48
>CR1	0.06 ± 0.02		
Stem cell source			
BM	0.08 ± 0.02	2.54 [0.98, 6.57]	0.06
Other	0.06 ± 0.02		
CMV constellation (patient/donor)			
+/−	0.07 ± 0.05	1.09 [0.41, 2.88]	0.70
Other	0.07 ± 0.02		
Recipient CMV status			
Negative	0.09 ± 0.02	1.66 [0.78, 3.53]	0.26
Positive	0.03 ± 0.02		

*BM* bone marrow, *CI* confidence interval, *CMV* cytomegalovirus, *CR1* first complete remisssion, *HSCT* hematopoietic stem cell transplantation, *MD* matched donor, *MMD* mismatched donor, *MSD* matched sibling donor, *SD* standard deviations, *yrs* years.

^a^Fine and Gray.

Bold values indicate *p*-value < 0.05.

In 58 of the 705 patients, a CNS irradiation boost was applied. Out of these, 23 were performed before, two after and 33 during HSCT conditioning. One of these 33 patients (3.0%) developed glioblastoma during follow-up, while three of the 525 (0.6%) other TBI patients without a recorded CNS radiation boost were diagnosed with glioblastoma (*p* = 0.22).

In summary, only TBI/VP-16 emerged as a significant risk factor for development of SMN in children with ALL in this cohort.

## Discussion

Several studies have found that while relapse-related mortality plateaus more than five years after HSCT in children, non-relapse mortality (NRM) from all causes increases during long-term follow-up, as does the cumulative incidence of SMN [10, 21]. In the prospective ALL-SCT-BFM-2003 trial, we found an accelerating increase of the cumulative incidence of SMN starting approximately five years after HSCT. SMN were exclusively observed in patients who had received TBI/VP16-based conditioning. Almost all previous studies on this subject were either retrospective in nature, included mainly adults, or contained a variety of conditioning regimens and/or diseases, as summarized in Supplementary Table 1. In contrast, we here present data from a prospective trial with uniform conditioning regimens exclusively in children with ALL.

The cumulative incidence of SMN was higher in our cohort than reported in most of the previous publications of large HSCT cohorts [11, 21–24]. In our study, TBI-based conditioning was the only statistically significant risk factor for the development of a SMN in univariate but not multivariate analysis. This may be due to the relatively small absolute number of SMN events, but we cannot exclude the rather unlikely possibility that other factors not considered in the univariate analysis were responsible for this effect. The negative effect of TBI had previously been observed in other studies [23, 25, 26]. One recent study retrospectively analyzed 670 children transplanted for ALL and AML. Consistent with our data, the authors found SMN exclusively in the TBI group with a cumulative incidence of >15% at 16 years in the TBI group [9]. The follow-up for these studies—including ours—is relatively short for the detection of the true incidence of SMN, which may occur 20 years or later after irradiation [27]. The patient cohort of Keslova et al. included cases of SMN up to 21.5 years after HSCT, which were diagnosed at a median of 11.7 years. Their observed final cumulative cancer incidence (only solid tumors) was 15.2% at 22 years with 90% of patients having received TBI [28]. Other risk factors, such as age of the recipient at HSCT and chronic GVHD, had been identified in other studies, but could not be reproduced in our cohort, possibly due to the relative low rate of chronic GVHD and the exclusion of very young patients from TBI in our study [21, 29].

It cannot be excluded that VP-16 was a factor in the development of SMN in our cohort. The most common SMN described after the use of topoisomerase inhibitors is secondary leukemia and MLL rearrangement. It can be caused by pulsed VP-16 exposure [30, 31]. Thus, VP-16 may be involved in the development of MDS of recipient origin. While rare, donor derived MDS has been described as a complication [32]. A potential mechanism could be therapy-related changes in the bone marrow niche [33]. In our cohort, secondary MDS of both donor and recipient origin occurred.

Potentially adding to the risk of SMN could be the anti-leukemic treatment prior to HSCT, but this information was not available for our analysis. Hijiya et al. show that ALL patients who stayed in first complete remission had cumulative incidences of SMN of about 4% at 15 years [34]. The omission of cranial irradiation seems to lower the risk of developing secondary brain tumors [35]. Patients in our study received fractionated TBI (6 × 2 Gy). This approach has been shown to result in fewer SMN [36]. This might be one contributing factor to the relatively low number of brain tumors in our study group, including in those with CNS radiation boosts. Better and more homogeneous organ-at-risk shielding, total lymphoid irradiation, or total marrow irradiation may also improve the long-term safety of TBI [37–41].

There are multiple putative strategies to improve the outcome of children with ALL after HSCT omitting TBI in the process, ideally to be tested in prospective randomized trials [42]. The multi-national FORUM alliance has demonstrated that this is feasible. One possible way to address this issue could be to reduce pre-HSCT leukemia burden without adding more potentially mutagenic chemotherapy, i.e. with new immune-based treatments such as blinatumomab or CAR-T cells [43–45]. Accepting a higher relapse risk after HSCT with chemotherapy-based conditioning may also potentially become acceptable, but only if effective and curative post HSCT therapies are available for relapsed patients. Currently it is unclear whether any of the new therapeutic approaches including CAR-T-cells can fulfil this promise, and historically post HSCT ALL relapse has a very poor prognosis [5, 46, 47]. Another possible approach could be to reduce the total TBI dose, instead building on the potent anti-leukemic efficacy of high dose VP-16 in this combination [48]. A conditioning regimen tailored to the risk of individual patients may also be an option. Patients with germline cancer predisposition may especially benefit from omitting radiation, and screening all patients by next generation sequencing will likely become standard practice soon. In our cohort, only one patient was retrospectively identified with inherited cancer predisposition syndrome after developing two SMN (colon carcinoma and glioblastoma), but standardized genetic screening for risk genes was not performed during our study.

This study underlines the importance of identifying modifiable risk factors. Careful long-term evaluation of patient cohorts with comparable risk factors and disease-specific screening programs and after-care are crucial. Information should be transferred to all involved clinical caretakers, which can be challenging because data protection is an important personal right. A potentially helpful tool, which could aid in gathering data in an accessible way, is the SurPass (‘Survivorship passport’) developed with the help from PanCare, the SIOP network, and parent and patient organizations [49]. For patients and families thorough screening and education about the risk for SMN is of the utmost importance. Potential additional risk factors such as smoking, incomplete HPV vaccination status and metabolic syndrome should be eliminated and health-promoting behavior should be encouraged [50, 51].

There are obvious limitations to our study, such as the lack of testing for cancer predisposition syndromes, and the relatively small size of the non-randomized comparator chemotherapy arm. The long-term follow up of the FORUM trial will address this question in a randomized manner in children >4 years of age. A longer follow-up period may result in more observed SMN in both the TBI and the non-TBI groups, and there may be a different latency of SMN occurrence in both groups. Nevertheless, we here report the first study with prospectively collected data from a uniform pediatric ALL cohort making its findings particularly relevant for current clinical practice in pediatric hematology/oncology and for long-term follow-up care.

Our findings may seem especially worrisome in light of recent study results of the FORUM trial. It shows that changing the conditioning regimen to a chemotherapy-based regimen in children with ALL > 4 years at HSCT is associated with increased relapse- and transplant-related mortality independent of immunophenotype, remission status, sex, age and pre-HSCT minimal residual disease (MRD) levels [5]. Whether other chemotherapy conditioning regimens or the addition of CAR T-cells to the pre HSCT treatment could be as effective as TBI for pediatric ALL, will have to be tested in carefully designed randomized trials [52]. On the other hand and importantly, 64% of the patients with SMN in our cohort were alive at a median follow-up of 5.1 years after diagnosis. All patients with glioblastoma succumbed to the disease, but half of the patients were diagnosed with thyroid cancer and all of them (but one with MDS as tertiary malignancy) survived, which is in line with the generally excellent OS of thyroid carcinoma [53].

Therefore, considering its leukemia-free survival benefit, the use of TBI as a gold standard conditioning regimen for children with ALL is still justifiable in our opinion. Careful extended long-term follow-up and individual cancer prevention strategies in high-risk patients are indicated. This large, prospective, multicenter trial confirms and quantifies the risk of SMN after HSCT in children with a TBI/VP-16 conditioning regimen. Future strategies to avoid or to optimize TBI in the conditioning of children with ALL will need to be tailored and prospectively studied in controlled trials in order to prevent unfavorable outcomes.

## Supplementary information

Supplementary data

## Data Availability

Original data are available upon reasonable request to Professor Christina Peters (christina.peters@stanna.at).
