# Supplementary material for: Incidence of subsequent malignancies after total body irradiation-based allogeneic HSCT in children with ALL – long-term follow-up from the prospective ALL-SCT 2003 trial
Source: Leukemia. 2022 Sep 12;36(11):2567–76. doi: 10.1038/s41375-022-01693-z (PMC9613465; doi:10.1038/s41375-022-01693-z)
Supplement: Supplementary file 1 — Supplementary data [file 41375_2022_1693_MOESM1_ESM.docx]

**Supplementary Material**

**Supplementary Table 1. Publications on subsequent malignancies (SMN) after allogeneic HSCT relevant for pediatric ALL patients** (2000 – 2021; excluding publications on patients with known genetic cancer predisposition).

| **First author; year; journal** | **Study type/ year of HSCTs/ Median FU after HSCT** | **Pediatric patients/ all patients**  **(n)** | **Underlying disease** | **Conditioning regimens** | **allo (n)/**  **auto (n)** | **Cumulative incidence of SMN at *n* years** | | | | | | **Number of SMN** | **Number of SMN after irradiation** | **Type of SMN and number of cases (n)** | **Identified risk factors for SMN (p < 0.05)** | **Additional information to study** |
| --- | --- | --- | --- | --- | --- | --- | --- | --- | --- | --- | --- | --- | --- | --- | --- | --- |
|  |  |  |  |  |  | 5 | | 10 | 15 | | 20 |  |  |  |  |  |
|  |  |  |  |  |  | additional information | | | | | |  |  |  |  |  |
| Socie; 2000; JCO^1^ | Inter-national BMT Registry database/  1964-1992/ 0.9 yrs (0 – 20.7) | 3,182/  3,182 | ALL, AML, undifferentiated AL | Radiation-based conditioning regime (87%); non-radiation based conditioning regime (13.1%) | allo (all) | 0.9% (CI  0.3-1.5%) | | 4.3% (CI 2.2-6.5%) | 11.0 % (CI 2.3-19.8%) | | n.a. | 46 | All solid tumors after TBI or CNS radiation | SCC (3), salivary gland mucoepidermoid carcinoma (2), osteosarcoma (2), MFH (1), Melanoma (4), thyroid papillary carcinoma (5), brain cancer (9), PTLDs (20) | High-dose TBI (≥ 10 Gy single-dose TBI or ≥ 13 Gy fractionated TBI), younger age at HSCT (<10 yrs) | Difference in risk by age at transplantation was confined to brain and thyroid cancers; decreased risk of solid tumors after chronic GvHD |
|  |  |  |  |  |  | For solid tumors only | | | | | |  |  |  |  |  |
| Favre-Schmuziger; 2000; BMT^2^ | Retro-spective cohort study/  1974 – 1996/ 7 yrs (1.5 – 23) | 387 (1 – 58 yrs of age; distribution n.a.) | ALL, AML, CML, SAA, other hem. diseases | Cy +/- VP-16 and TBI; Bu + Cy; Cy alone | allo (all) | n.a. | | n.a. | 9% (CI  1-17% CI) | | n.a. | 6 in 5 pts | n.a. | Endometrial carcinoma (1), carcinoma of the thyroid gland (1), cervical carcinoma (1), sarcoma of the small intestine (1), osteosarcoma (1), ovarian carcinoma (1) | n.a. | Study only looked at secondary solid tumors; 3 of the tumors in 2 patients with HSCT <19 yrs |
|  |  |  |  |  |  | For solid tumors only | | | | | |  |  |  |  |  |
| Bhatia; 2001; JCO^3^ | Retro-spective cohort and nested case-control study/ 1976 – 1998/ 3.3 yrs (0.1 – 21.1) | 2,129 (age 1-75 years at HSCT; distribution n.a.) | ALL, AML, CML, SAA, lymphoma | Cy, VP-16, Bu and/or TBI | allo (1,370)/ auto (759) | 1.6 ± 0.5% | | 6.1 ± 1.6% | n.a. | | n.a. | 29 | 15 (TBI), 5 (no-TBI)  (nonmelanoma skin cancer excluded from this analysis) | Nonmelanoma skin cancer (9), cervix uterui carcinoma (4), salivary gland (3), SCC (4), breast (2), liver (2), thyroid (2), astrocytoma (1), MFH (1), synovial carcinoma (1) | Age younger than 34 yrs at time of HSCT; TBI with higher risk of liver or thyroid cancer development | All patients with SCC had cGVHD; study only looked at secondary solid tumors |
|  |  |  |  |  |  | For solid cancers | | | | | |  |  |  |  |  |
|  |  |  |  |  |  |  | |  | solid tumors: 5.6% ± 2.2% at 13 yrs | |  |  |  |  |  |  |
| Baker; 2003; JCO^4^ | Prospec-tively collected database/  1974 – 2001/ 5 yrs (0.5 – 25) | 1,514 (<20 yrs)/ 3,372 | AA, immune deficieny, storage disorder, ALL, AML, CML, MDS, NHL, HL, NB, breast Ca, other | TBI/TLI (78%) ± Cy or with other chemotherapy agents; chemotherapy-only regimens (22%) | allo (2,179)/ auto (1,193) | 2.3%  (CI 1.8 – 2.8%) | | 4.0% (CI 3.2 – 4.8) | - | | 5.6%  (CI 2.2 – 5.4%) | 147 (in 137 pts; 50 in pts < 20 yrs) | n.a. | Adenocarcinoma (4), basal cell carcinoma (11), breast (4), carcinoid (1), carcinoma-in-situ (5), carcinoma (unknown primary, 2), CML (1), brain tumor (4), Hodgkin’s disease (4), MDS/AML (36), melanoma (8), mucoepidermoid parotid (3), neuroblastoma (1), thyroid (1), PTLD (44), renal cell carcinoma (1), sarcoma (6), SCC (11) | 60-fold higher risk for SMN for age <10 yrs at HSCT | No Increased risk of solid tumors in patients who received TBI (p=0.27) |
|  |  |  |  |  |  | Excluding PTLD | | | | | |  |  |  |  |  |
| Cohen;  2007; JCO^5^ | Retro-spective investi-gational study/  1985 – 2003/ 12.7 yrs (25^th^ percentile 10.3; 75^th^ percentile 16.5) | 18,946 (< 21 years)/  70,859 | SAA, hem. cancer, solid tumor, other | TBI/TAI (55.1%), | allo (31,771  /auto (38,988/ missing (100) | - | | - | - | | - | 35 (22 < 21 yrs;); 21 in allo group | 24/35 with TBI/TAI | Thyroid carcinoma (35) | Young age at transplantation (RR 4.8 at 11 to 20 yrs; RR 20.4 , <11 yrs); TBI/TAI (RR 3.44); female gender (RR 2.8); chronic GVHD (RR 2.94) | Only evaluating secondary thyroid carcinoma |
| Rizzo; 2009; Blood^6^ | Cohort Study/  1994 – 1996/ 6,641 pts. Followed > 5 yrs; 1,985 > 10 yrs; 378 > 15 yrs | 9.831 (<20 yrs)/ 28,874 | ALL, ANLL, CML, other leukemia, NHL, HL, myeloma, SAA, MDS, MPD, hemoglobinopathies, other | TBI (67%) | allo (all) | - | | 2.5% (CI 2.0-3.0%) | 5.8% (CI 4.3-7.0%) | | 8.8% (CI 6.2-12.3%) | 153 | 125 (TBI), 9 (only LFI) | Oral cavity and pharynx (27), esophagus (3), colon (2), rectum/anus (6), liver (7), bronchus/lung (8), female breast (13), female genital system (7), testis (3), melanoma (18), CNS (18), thyroid (16), bones (6), soft tissue (7), other (12) | Irradiation (O/E 2.68 versus 1.26, p=0.001), particularly high risk in children irradiated <10 yrs (55-fold risk increase); increased risk for SCCs in chronic GVHD (RR 5.04) | Only looking at solid cancers; GVHD in 56 of patients with SMN |
|  |  |  |  |  |  | For solid cancers | | | | | |  |  |  |  |  |
| Ricardi; 2009; Strahlentherapie und Onkologie^7^ | Cohort Study/  n.a./ 8.6 yrs (5.1 – 17.9) | 51 (2-16.4 yrs at HSCT; distribution n.a.) | ALL, AML, CML, NHL | TBI (100%) + different chemotherapies | allo (32)/ auto (19) | n.a. | | 10% (SE=0.07) | n.a. | | n.a. | 3 | 3 (TBI) | Thyroid carcinoma (3) | n.a. | Only patients with disease-free survival at 5 yrs post/HSCT included |
| Bresters; 2010; BMT^8^ | Cohort study/  n.a./ 7.2 yrs (2.0 – 21) | 162/  162 | Hem.-oncological disease, non-malignant hem. disease, immune deficiencies, other inborn errors of metabolism | TBI/TAI (48%); all regiments including Bu and Cy with or without other cytotoxic drugs (but no TBI; 45%); other regimens (including reduced intensity conditioning; 8%) | allo (all) | n.a. | | n.a. | n.a. | | n.a. | 1 | n.a. | Malignant melanoma | Patient with cGVHD (no statistics due to single affected patient) | Study was assessing cumulative incidence and severity of late effects after HSCT in childhood (no focus on SMN); only pts that survived at least 2 yrs after HSCT |
| Omori; 2013; Springer Plus^9^ | Retro-spective (BMT data-base)/  1995 – 2010/ 10.5 yrs for only survivors (max. 16.4) | 50/  370 | ALL, AML, MDS, CML | TBI + Cy | allo (all) | 2.2 ± 1.2 % | | 6.5 ± 2.8% | n.a. | | n.a. | 11 in 10 pts | all pts in the study conditioned with TBI | Thyroid (1), sub-maxilary gland tumor (1), esophageal cancer (2), oral cavity carcinoma (1), gastric cancer (2), ureteral cancer (1), border malignant ovarian tumor (1), extragonadal germ cell tumor (1), head and neck cancer (1) | No comparison of different potential risk factors shown | Only 2 tumors in the pediatric population (oral cavity carcinoma, gastric cancer) |
| Nelson; 2015; Leukemia^10^ | ABMTRR Report/  1982 – 2007/ 8.7 yrs (2.0 – 25.9) | 717 (all <15 yrs)/ 717 | ALL, AML, NHL, MDS/JMML/ other MPD, CML, HL | Radiation-based (59%); nonradiation-based (39%); no conditioning (0.1%); unknown (1%) | allo (all) | 0.8 ± 0.4% | | 2.4 ± 0.7% | 3.3 ± 1.0% | | 8.7 ± 2.9% at the end of follow-up | 17 | 94% (TBI) | thyroid (8), brain (4), parotid gland (1), colon (1), connective and soft tissue (1), scrotum (1), unspecified site (1) | TBI; increased risk for NHL as primary diagnosis | Also gathered data regarding pre-HSCT treatment (radiation in 24%) |
| Bresters;  2016; BMT^11^ | Multi-center retro-spective study/  1987-2011/ 10.7 yrs (5.0 – 25.3) | 840/  840 (all < 3 yrs at HSCT) | AML, ALL, JMML/MPD, MDS, CML, Lymphoma | TBI (25%); chemotherapy only (75%) | allo (all) | 0.01 ± 0.01 | | 0.02 ± 0.01 | 0.06± 0.02 | | 0.08 ± 0.03 | 13 | n.a. | Thyroid carcinoma (4), basal cell carcinoma (3), melanoma (1), brain tumour (2), osteosarcoma (1), ALL (1), AML (1) | TBI as risk factor for all the long term effects studied (thyroid dysfunction, growth retardation, delayed pubertal development, SMN) | only analyzed patients that survived more than 5 yrs after HSCT |
| Gündüz;  2017;  Clinical Transplantation^12^ | Single center retro-spective study/  1988 – 2015/ 1.6 yrs (0 – 26.8) | 979 (age 5-71 yrs at HSCT; distribution n.a.) | AML, ALL, CML, CLL, NHL, HL, MDS, MPD, plasma cell disease, AA, other | Bu + Cy (57%); Cy + TBI (20%); Flu + Cy + TBI (2); FluBu (6%); other chemotherapy based (15%) | allo (all) | 1.3 ± 0.5% | | 3.9 ± 1.2% | n.a. | | n.a. | 15 | 2 (TBI) | PTLD (3), Head and neck (6), breast (1), adrenal cortex (1), pancreas (1), lung (1), esophagus (1), sarcoma (1) | Benign hematological disease as HSCT indication; cGVHD; ATG in conditioning regimen |  |
| Hierlmeier;  2018; Plos one^13^ | Retro-spective analysis/  2005 – 2015/ 2.3 yrs (± 2.6) | 229/  229 | Leukemia, lymphoma, solid tumor, CNS tumor, other | n.a. | allo (126)/ auto (103) | n.a. | | n.a. | n.a. | | n.a. | 1 (allo) | n.a. | MDS (1) | n.a. | Irradiation in pre-HSCT treatment in 34% of the pts, looking at variety of early and late complications after HSCT |
| Holmqvist; 2018; JAMA Oncology^14^ | Retro-spective cohort study/  1974 – 2010/ 14.9 yrs (2.0 – 41.2) | 1388/  1388  (0 – 21 yrs) | ALL, AML/ MDS, SAA, inborn errors of metabolism | TBI (64%); Cy (81%), Bu (26%) | allo (all) | n.a. | | n.a. | n.a. | | n.a. | 45 | n.a. | n.a. | TBI as risk factor for late mortality (in general) | This study only looked at the causes of late mortality (> 2 yrs after HSCT), not at the total number of SMN; 45/295 deceased patients died due to SMN |
| Wareham; 2019;  J Cancer Res Clin Oncol^15^ | Cohort study/  2004 – 2014/ 2.6 yrs (0.8 – 5.4) for HSCT | 237/  992 | Malignant disease (90%), non-malignant disease or unknown (10%) | n.a. | allo (all) | Standardized incidence ratio of cancer after HSCT compared to Danish population: 2.18 (1.57-2.96) | | | | | | 75 | n.a. | n.a. | Cancer associated with older age (>50 bs <20 yrs), umbilical cord blood transplant and > 1 HSCT; TBI | Study looks at both HSCT (992 pts) and solid organ transplantation (1656 pts) |
| Tichelli; 2019; JAMA Oncology^16^ | EBMT cohort study/  2000 – 2014/ n.a. | 220,617 🡪 4,065 with SMN | n.a. | n.a. | allo (1,443)/  Auto (2,622) | n.a. | | n.a. | n.a. | | n.a. | 4,065 | n.a. | Allo (1,443): thyroid (78), cervix (35), prostate (93), breast (196), melanoma (160), kidney (47), oropharyngeal (104), bladder (31), ovarian (26), sarcoma (96), colorectal (121), endometrial (26), gastric (57), brain (71), esophageal (53), hepatobiliary (32), lung (179), pancreas (36), n.a. (2) | n.a. | Only including patients with SMN into the study (4,065/220,617); only looking at solid cancers and at risk of death (not at risk of SMN) |
| Saglio;  2020;  BMT^17^ | Retro-spective multi-center study/  2000 – 2012/  7 yrs (5 – 16) | 670/  670  (all 3-18 yrs) | ALL, AML | TBI-based (70%); busulfan-based (30%) | allo (all) | ~ 0.5% (TBI); 0% (Bu) | | ~ 2.7% (TBI); 0% (Bu) | ~ 18% (TBI); 0% (Bu) | | n.a. | 25 | 25 (TBI) | Thyroid (3), malignant melanoma (2), renal carcinoma (1), grade IV glioma (1), undifferentiated sarcoma (2); neurofibroma (1), HCC (1), AML (1) | TBI; ALL (compared to AML) | No SMN in Bu group;  no SMN after HSCT for AML;  only patients analyzed that survived more than 5 years after HSCT; looking at different long-term effects (not only se. mal.) |
| Keslova; 2020; Neoplasma^18^ | Retro-spective single center/  1989 – 2017/ 9.9 yrs (1.0 – 29.8) | 426/  426 | ALL, AML, MDS, CML, immune deficiency, metabolic disease, SAA, osteopetrosis, other | TBI-based (34.3%), Non-TBI-based (65.7%) | allo (all) | Cumulative incidence 15.2 ± 3.9% at 22 yrs post-HSCT (TBI: 34.7 ± 8.9%; Non-TBI: 1.5 ± 1.1%) | | | | | | 20 (prim diagnosis: ALL in 13, AML in 3, MDS in 2, SAA in 2) | 18 (TBI-based) | Thyroid cancer (9), oral cavity cancer (3), melanoma (3), malignant schwannoma (2), peritoneal mesothelioma (1), basal cell carcinoma (1), breast cancer (1) | TBI | only analyzed patients that survived more than 1 yr after HSCT; pts with known genetic cancer predisposition were excluded |
| Bhatia; 2021; JAMA Oncology^19^ | Cohort multi-center study/ 1974 – 2014/ 12 yrs (2.0 – 44.0) | 1,343/  4,741 | AML, ALL, CML, MDS, NHL, SAA, other | TBI-based (61.3%), Non-TBI-based (38.1%), missing (0.6%) | allo (all) | n.a. | n.a. | | n.a. | 7.0% at 30 years | | 390 observed deaths due to SMN | n.a. | n.a. | Chronic GvHD (HR 1.88, CI 1.1-3.2); Transplant Year (2005-2014 0.31, CI 0.1-0.8), older age (>45 yrs) at HSCT (3.42, CI 1.7-6.9) | only analyzed patients that survived more than 2 yrs after HSCT; not looking at SMN, but general trends in late mortality and life expectancy |
| Santarone; 2021; Bone Marrow Transplantation^20^ | Retro-spective study/ 1977-2016/  17 yrs (3 – 38) | 254/  908 | AML, ALL, CML, MDS, NHL, HL, SAA, immune deficiencies, hematopathies, other | TBI-Based (24%), Non-TBI-based (76%)/ MAC (84%), RIC (16%) | allo (all) | Cumulative incidence 3.5% at 35 yrs post-HSCT (CI 0.666-14) for secondary oral cancer | | | | | | 12 (3 in pediatric HSCT pts) | 1 (TBI-based; 2 with irradiation before HSCT) | Secondary oral cancer | RIC (2.1% versus 11.2% after MAC, p=0.026), oral cGVHD duration ≥ 15 months (p<0.001) | only looking at secondary oral cancer |

AA=aplastic anemia, ABMTRR=Australasian BMT Recipient Registry, AL=acute leukemia, ALL=acute lymphoblastic leukemia, allo=allogeneic, AML=acute myeloid leukemia, auto=autologous, BMT= bone marrow transplant(ation), Bu=busulfan, cGVHD=chronic graft versus host disease, CML=chronic myelogenous leukemia, CNS=central nervous system, CI=confidence interval, Cy=cyclophosphamide, Flu=fludarabine, FU=follow-up, Gy=gray, HCC=hepatocellular carcinoma, hem.=hematological, HL=Hodgkin lymphoma, HSCT=hematopoietic stem cell transplantation, J Cancer Res Clin=Oncol Journal of Cancer Research and Clinical Oncology, JCO=Journal of Clinical Oncology, JMML=juvenile myelomonocytic leukemia, LFI=limited field irradiation, MAC=myeloablative conditioning, MDS=myelodysplastic syndrome, MFH=malignant fibrous histiocytoma, MPD=myeloproliferative disease, n.a.=not available, NB=neuroblastoma, NHL=Non-Hodgkin lymphoma, pts=patients, PTLD=posttransplant lymphoproliferative disorder, RIC=reduced intensity conditioning, SAA=severe aplastic anemia, SCC=squamous cell carcinoma, SMN=subsequent malignant neoplasm/s, TAI=thoraco-abdominal irradiation, TBI=total body irradiation, VP-16=etoposide, yrs=years.

**References**

1. Socie G, Curtis RE, Deeg HJ, et al. New malignant diseases after allogeneic marrow transplantation for childhood acute leukemia. *J Clin Oncol*. 2000;18(2):348-357.

2. Favre-Schmuziger G, Hofer S, Passweg J, et al. Treatment of solid tumors following allogeneic bone marrow transplantation. *Bone Marrow Transplant*. 2000;25(8):895-898.

3. Bhatia S, Louie AD, Bhatia R, et al. Solid cancers after bone marrow transplantation. *J Clin Oncol*. 2001;19(2):464-471.

4. Baker KS, DeFor TE, Burns LJ, Ramsay NK, Neglia JP, Robison LL. New malignancies after blood or marrow stem-cell transplantation in children and adults: incidence and risk factors. *J Clin Oncol*. 2003;21(7):1352-1358.

5. Cohen A, Rovelli A, Merlo DF, et al. Risk for secondary thyroid carcinoma after hematopoietic stem-cell transplantation: an EBMT Late Effects Working Party Study. *J Clin Oncol*. 2007;25(17):2449-2454.

6. Rizzo JD, Curtis RE, Socie G, et al. Solid cancers after allogeneic hematopoietic cell transplantation. *Blood*. 2009;113(5):1175-1183.

7. Ricardi U, Filippi AR, Biasin E, et al. Late toxicity in children undergoing hematopoietic stem cell transplantation with TBI-containing conditioning regimens for hematological malignancies. *Strahlenther Onkol*. 2009;185 Suppl 2:17-20.

8. Bresters D, van Gils IC, Kollen WJ, et al. High burden of late effects after haematopoietic stem cell transplantation in childhood: a single-centre study. *Bone Marrow Transplant*. 2010;45(1):79-85.

9. Omori M, Yamashita H, Shinohara A, et al. Eleven secondary cancers after hematopoietic stem cell transplantation using a total body irradiation-based regimen in 370 consecutive pediatric and adult patients. *Springerplus*. 2013;2:424.

10. Nelson AS, Ashton LJ, Vajdic CM, et al. Second cancers and late mortality in Australian children treated by allogeneic HSCT for haematological malignancy. *Leukemia*. 2015;29(2):441-447.

11. Bresters D, Lawitschka A, Cugno C, et al. Incidence and severity of crucial late effects after allogeneic HSCT for malignancy under the age of 3 years: TBI is what really matters. *Bone Marrow Transplant*. 2016;51(11):1482-1489.

12. Gunduz M, Ozen M, Sahin U, et al. Subsequent malignancies after allogeneic hematopoietic stem cell transplantation. *Clin Transplant*. 2017;31(7).

13. Hierlmeier S, Eyrich M, Wolfl M, Schlegel PG, Wiegering V. Early and late complications following hematopoietic stem cell transplantation in pediatric patients - A retrospective analysis over 11 years. *PLoS One*. 2018;13(10):e0204914.

14. Holmqvist AS, Chen Y, Wu J, et al. Assessment of Late Mortality Risk After Allogeneic Blood or Marrow Transplantation Performed in Childhood. *JAMA Oncol*. 2018;4(12):e182453.

15. Wareham NE, Li Q, Sengelov H, et al. "Risk of de novo or secondary cancer after solid organ or allogeneic haematopoietic stem cell transplantation". *J Cancer Res Clin Oncol*. 2019;145(12):3125-3135.

16. Tichelli A, Beohou E, Labopin M, et al. Evaluation of Second Solid Cancers After Hematopoietic Stem Cell Transplantation in European Patients. *JAMA Oncol*. 2019;5(2):229-235.

17. Saglio F, Zecca M, Pagliara D, et al. Occurrence of long-term effects after hematopoietic stem cell transplantation in children affected by acute leukemia receiving either busulfan or total body irradiation: results of an AIEOP (Associazione Italiana Ematologia Oncologia Pediatrica) retrospective study. *Bone Marrow Transplant*. 2020;55(10):1918-1927.

18. Keslova P, Formankova R, Riha P, et al. Total body irradiation is a crucial risk factor for developing secondary carcinomas after allogeneic hematopoietic stem cell transplantation in childhood. *Neoplasma*. 2020;67(5):1164-1169.

19. Bhatia S, Dai C, Landier W, et al. Trends in Late Mortality and Life Expectancy After Allogeneic Blood or Marrow Transplantation Over 4 Decades: A Blood or Marrow Transplant Survivor Study Report. *JAMA Oncol*. 2021;7(11):1626-1634.

20. Santarone S, Natale A, Angelini S, et al. Secondary oral cancer following hematopoietic cell transplantation. *Bone Marrow Transplant*. 2021;56(5):1038-1046.

**Supplementary Table 2. Multivariate analysis of risk factors for developing a subsequent malignancy (SMN).**

| **Parameter** | ***p*-value^1^** | **Hazard ratio (HR)** | **95% HR confidence limits** |
| --- | --- | --- | --- |
| **conditioning** |  |  |  |
| TBI vs Non-TBI conditioning regimen | 0.196 | 6.74 | [0.37, 121.89] |
| **age at HSCT** |  |  |  |
| Age ≤ 10 yrs vs other | 0.190 | 1.72 | [0.77, 3.84] |
| **donor type** |  |  |  |
| MD vs MSD | 0.205 | 1.78 | [0.73, 4.33] |
| MMD vs MSD | 0.78 | 1.32 | [0.19, 9.00] |
| **remission status at HSCT** |  |  |  |
| > CR1 vs other | 0.77 | 1.13 | [0.50, 2.59] |
| **stem cell source** |  |  |  |
| BM vs other | 0.40 | 1.54 | [0.56, 4.22] |
| **CMV constellation (recipient/donor)** |  |  |  |
| recipient + / donor – vs other | 0.53 | 1.40 | [0.49, 4.03] |
| **ALL phenotype** |  |  |  |
| B-cell vs other | 0.23 | 0.60 | [0.25, 1.40] |
| **Gender** |  |  |  |
| Recipient male/ donor female vs other | 0.09 | 2.05 | [0.90, 4.66] |

BM bone marrow; CMV cytomegalovirus; CR1 first complete remisssion; HSCT hematopoietic stem cell transplantation; MD matched donor; MMD mismatched donor; MSD matched sibling donor; TBI total body irradiation (conditioning regimen); vs versus; yrs years.

**Supplementary Figure 1**


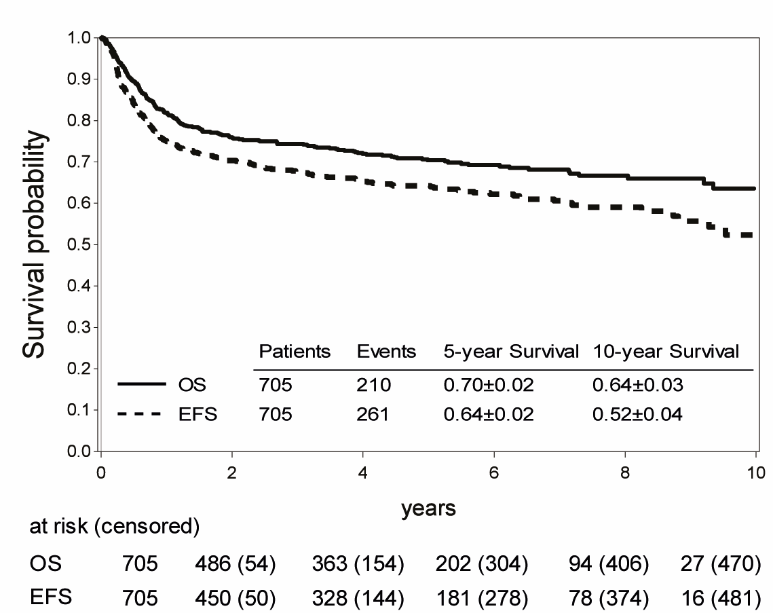


**Figure S1: Overall survival (OS, solid line) and event-free survival (EFS, dashed line)** after HSCT. A more detailed description of outcome of the TBI and non-TBI cohorts has previously been published in Peters et al (J Clin Oncol 22:1265-74, 2015).

**Other supplementary material:**

**Study protocol**

The original study protocol (ALL SZT-BFM 2003) in German language is available upon written request to the corresponding author at [malbert@med.lmu.de](mailto:malbert@med.lmu.de) .
